# Mechanism of Generation of ZnO Microstructures by Microwave-Assisted Hydrothermal Approach

**DOI:** 10.3390/ma6062497

**Published:** 2013-06-18

**Authors:** Ravish Majithia, Jeffrey Speich, Kenith E. Meissner

**Affiliations:** 1 Department of Materials Science and Engineering, Texas A&M University, College Station, TX 77843, USA; E-Mail: ravishmajithia@tamu.edu; 2 Department of Biomedical Engineering, 5045 ETB, Texas A&M University, TX 77843, USA; E-Mail: kmeissner@tamu.edu

**Keywords:** microwave-assisted, zinc oxide, hydrothermal, hemimorphite

## Abstract

In this report, a technique for rapid synthesis of ZnO microstructures by microwave-assisted heating of precursors at hydrothermal conditions is demonstrated. Further, the reaction mechanism for the growth of ZnO microstructures is analyzed. An accelerated rate of reaction obtained using microwaves enables a dissolution-recrystallization mechanism for generation of one dimensional (1D) rod-like structures, thereby showing that time of reaction can be used to dictate ZnO microstructure morphology.

## 1. Introduction

ZnO possesses a very rich family of micro- and nano-structures owing to numerous methods for synthesis including vapor phase processes, such as thermal evaporation [1] and chemical vapor deposition (CVD) [2], as well as solution-based processes, such as aqueous hydrolysis [3,4] and electrochemical reactions [5]. Each synthesis technique has a unique set of advantages and disadvantages. Solution-based techniques, which typically include chemical heating baths, offer a low-cost benchtop alternative to vapor phase processes and are more attractive for commercial scale-up. Aqueous-based synthesis techniques in particular are advantageous over vapor phase techniques as they offer the ability to introduce structural variation in ZnO micro- and nano-structures by means of additives such as hexamethylenetetramine (HMT) [3,6,7,8,9], cetyltrimethylammonium bromide [10] and ethylenediamine [11].

A key disadvantage to aqueous-based techniques, typically carried out in chemical baths, is the long time scale required for synthesis, typically spanning hours. This is adverse for commercialization and also compromises the quality of the resulting ZnO crystals by introducing defects [12]. Despite being very versatile, aqueous-based reaction systems for ZnO structure synthesis exhibit a fairly complex relationship between reaction mechanism and reaction parameters controlling structural morphology. Thus, an aqueous-based synthesis recipe for ZnO micro- and nano-structures demands a high degree of reaction parameter control to accurately produce the desired structural morphology.

As an alternative to chemical baths, microwave-assisted heating can drastically reduce the time required for synthesis of ZnO micro- and nano-structures [13,14,15,16,17,18]. Microwave irradiation heats a substance by dipole polarization and ionic conduction. It thus interacts with reaction mixtures on a molecular level leading to an accelerated rate of reaction and shortened reaction time. Microwave-assisted approaches can also provide a high degree of morphological control during the synthesis of ZnO microstructures by exerting an almost instantaneous and dynamic control over reaction temperature.

Reaction parameters and design motifs for microwave-assisted synthesis in solution-phase reactions would significantly differ from convective methods. For example, since microwaves heat a substance by dipole polarization, reaction mixtures in polar solvents, like water or alcohol, heat more rapidly than those in apolar solvents like toluene. In addition, presence of salts in reaction mixtures affects the rate of heating, in that, mixtures with higher salt concentrations heat faster. Such effects can potentially lead to alternative reaction mechanisms unobserved in convective heating methods and can significantly affect microstructure product morphology. While the use of microwaves has obvious advantages, in terms of process parameter control and shorter reaction times, detailed studies elucidating the reaction mechanism for synthesis of ZnO structures remain to be done.

In this work ZnO microstructures have been synthesized in a single-mode microwave reactor, a CEM Discover^®^ system (CEM Corp., Matthews, NC, USA), equipped with an Intellivent pressure device via alkaline hydrolysis under hydrothermal conditions. The microwave reactor consists of a circular cavity, containing a waveguide, which delivers single-mode microwaves for uniform sample heating without any hot or cold spots in the reaction vessel that are typical for a domestic multimode microwave oven. The single-mode microwave cavity is designed to provide a higher energy density per unit volume of the sample allowing for an efficient preparative chemistry. A detailed study of the the mechanism of synthesis of ZnO microstructures, within controlled parameters, by a microwave-assisted hydrothermal approach is undertaken in this work.

## 2. Experimental Section

The mechanism of synthesis of ZnO microstructures, generated with microwave-assisted heating under hydrothermal conditions, is investigated in this report. For this, analytical reagent grade zinc nitrate hexahydrate (Zn(NO_3_)_2_·6H_2_O, 99%, Sigma-Aldrich) and HMT (C_6_H_12_N_4_, 99%, Sigma-Aldrich) were used in all experiments. One milliliter of each precursor, at 25 mM concentration, was mixed and heated in a 10 mL glass vessel capable of withstanding pressures of up to 300 psi. The vessel was placed in the single mode microwave cavity of the CEM Discover^®^ system. A moderate precursor concentration at 25 mM was chosen for study after consulting literature on the generation of ZnO microstructures using convectional-heating since they tend to promote the formation of rod-like microstructures [19]. The mixture of Zn(NO_3_)_2_ and HMT was heated up to 170 °C, the maximum allowable safe temperature for this reaction system, under a pressure of 100–150 psi for times ranging from 2 to 20 min. The ramping times required for samples to heat, ~100 s for 170 °C, are not included in the hold times reported (Figure S1). Predetermined temperature and pressure set points in the reaction system are dynamically maintained by means of microwave power used during heating determined by a PID controller. A contactless IR sensor and the Intellivent pressure device, each of which are integrated in the CEM Discover^®^ system, are used to monitor temperature and pressure respectively in the reaction vessel. Samples were subsequently cooled, after desired heating time, using a compressed air flow around the reaction vessel. The resulting product ZnO microstructures were centrifuged and washed once with methanol before morphological and chemical characterization using Scanning Electron Microscopy (SEM), including Energy Dispersive X-Ray Spectroscopy (EDS) and Powder X-Ray Diffraction (XRD).

## 3. Results and Discussion

**Figure 1:**
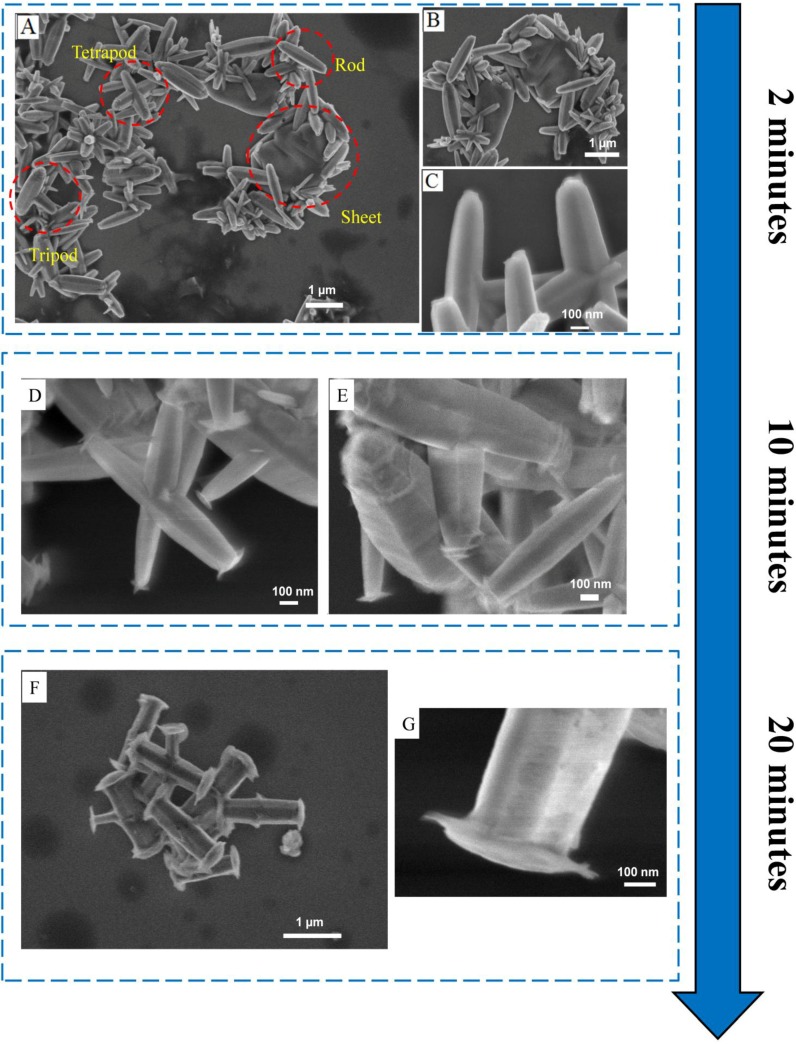
SEM images of ZnO microstructures formed by heating 25 mM of an equimolar mixture of HMT:Zn^2+^ at 170 °C, for various times, in a microwave-assisted reaction (**A**–**C**) A large variety of microstructures are generated at short reaction times of 2 min; (**D**,**E**) At longer synthesis times of 10 min only tripods and tetrapods are observed; (**F**,**G**) A mixture of rods and tripods is observed after heating for a period of 20 min.

ZnO microstructures undergo a gradual morphological evolution over 20 min of reaction time as shown in Figure 1. The morphological evolution of ZnO microstructures for various times ranging from 2 to 20 min, synthesized under the experimental conditions described above, occurs as a gradual continuum with representative images shown in Figure 1 at 2, 10, and 20 min reaction times. Microstructures synthesized at reaction times of two minutes exhibit large variations in morphology (Figure 1A–C), which includes irregular sheet-like structures and rods (Figure 1B) as well as tripods and tetrapods (Figure 1C). The variations in morphology seen in ZnO microstructures at short reaction times of two minutes are reduced at longer times of 10 and 20 min. At reaction times of 10 min, only tetrapods and tripods are observed, as seen in Figure 1D,E, respectively. At reaction times of 20 min, a mixture containing tripods and a large proportion of rods is generated, as seen in Figure 1F. The ZnO microrods formed after 20 min of reaction time are 1.45 ± 0.1 µm long and 0.38 ± 0.06 µm in diameter.

The decrease in the complexity and variety of ZnO microstructures is accompanied by an initiation of cap formation along the longer axes of rods and tripods. Distinct caps perpendicular to the longest axes of rods, with diameters slightly larger than that of the rods themselves (0.5 ± 0.08 µm), are observed on microstructures generated after 20 min of reaction time, as seen in Figure 1G. Such cap formation is gradual, and caps are not observed on tripods and tetrapods generated after two minutes of reaction time, as seen in Figure 1C. Caps are also not as well developed for structures obtained after 10 min of heating as they are for the ones obtained after 20 min.

### 3.1. Discussion of ZnO Microstructure Morphology

The effect of an accelerated rate of reaction in a microwave-assisted system manifests itself in the evolution of structural morphologies as seen in Figure 1. Such variation in microstructure morphology, over just 20 min of reaction time, has not been reported with convectional-heating methods. Additionally, the morphological evolution of the ZnO microstructures is accompanied by a unique, gradual cap-like formation along the longer axes of rods and tripods. Caps are not observed on microstructures during initial the phase of the reaction (Figure 1C) but are well developed on microstructures obtained after 20 min (Figure 1G).

**Figure 2:**
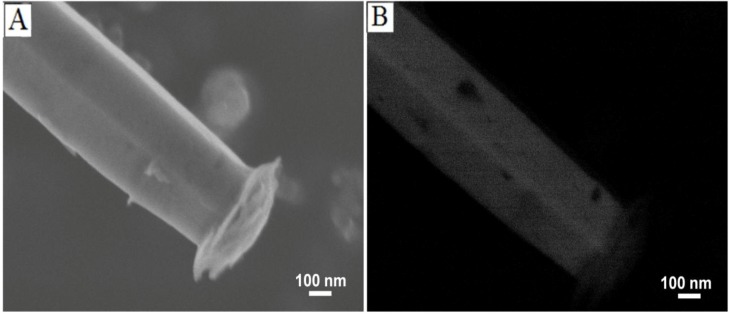
SEM of a ZnO microrods obtained using secondary electrons (**A**) and backscattered electrons; (**B**) show a difference in contrast between the microrod and the cap indicating a difference in electron density or crystal phase.

SEM images using secondary and backscattered electrons, as seen in Figure 2A,B, respectively, show a marked difference in relative contrast between the ZnO microrods (obtained after 20 min of heating) and the caps on their ends. In SEM, images generated by detection of secondary electrons, which are ejected within a few nanometers from the sample surface due to inelastic scatter, show contrast based on morphological features offering high resolution. On the other hand, images generated using backscattered electrons, which originate from the volume of the sample, exhibit contrast between areas with different chemical compositions. Areas containing heavy elements with high atomic numbers generate backscattered electrons more strongly than light elements (*i.e.*, low atomic number) and thus appear brighter on an image generated by backscattered electrons [20]. Thus, the differential contrast between the caps and microrods on the image generated using backscattered electrons (Figure 2B) indicates that the caps have a different chemical composition.

**Figure 3:**
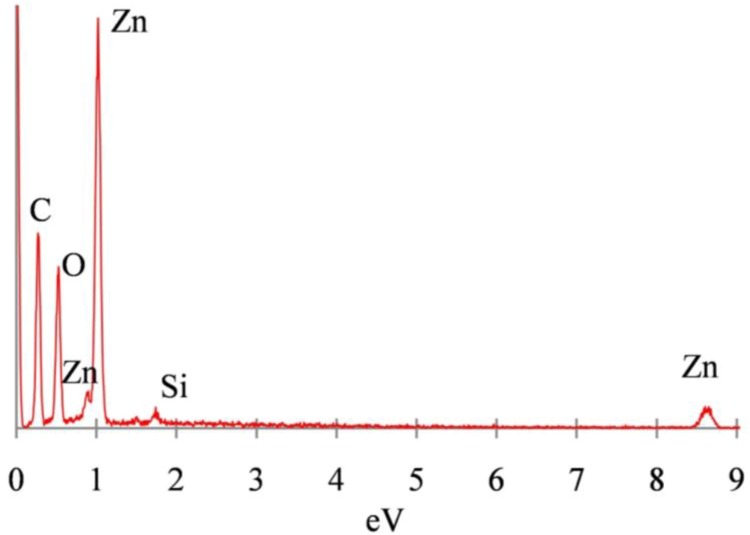
Energy Dispersive X-Ray Spectroscopy (EDS) spectrum for ZnO microstructures generated at 20 min of reaction time showing presence of Si.

The chemical composition of ZnO microstructures, generated after 20 min of reaction time, can be further analyzed by EDS and Powder XRD. EDS, a complementary technique in SEM which detects energies of X-rays generated by a sample upon excitation with an electron beam, can be used for elemental analysis and mapping of the ZnO microstructures [20]. EDS of ZnO microstructures generated at 20 min of reaction time qualitatively shows the presence of trace amounts of Si inside the ZnO microstructures, as seen in Figure 3. The peak corresponding to carbon, seen in Figure 3, is attributed to the carbon tape used during sample preparation. A quantitative analysis and elemental mapping of the ZnO microrods is challenging owing to the low resolution (~1 µm) of the EDS detector in the JEOL JSM-7500 FE-SEM used in this work. EDS can also be performed in conjunction with Transmission electron microscopy (TEM) and would typically offer better resolution for elemental mapping. However, TEM is not feasible for microstructures with diameters greater than 100 nm.

**Figure 4:**
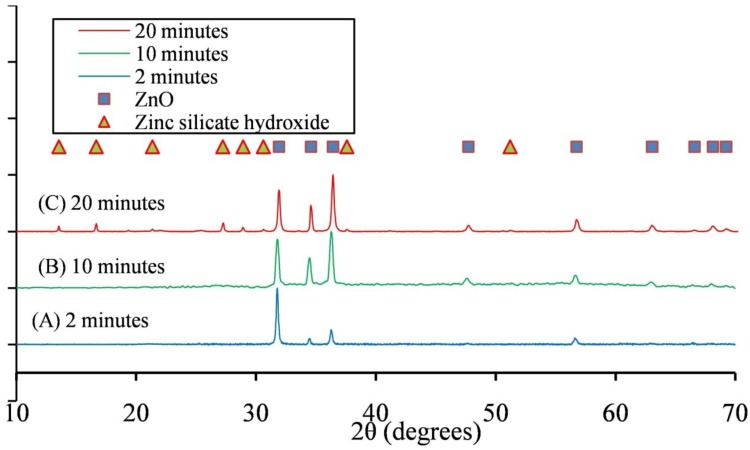
Powder X-Ray Diffraction (XRD) plots of ZnO microparticles generated with microwaves for a period of (**A**) 2; (**B**) 10; and (**C**) 20 min.

Powder XRD of ZnO microstructures confirms the presence of Si in ZnO microstructures generated at 20 min of reaction time. Powder XRD fingerprinting, used in this work, can provide information about crystal structure and chemical composition of the desired sample [20]. For XRD measurements, a Bruker D8 Bragg-Brentano diffractometer (CuKα radiation; 40 kV, 40 mA) fitted with LynxEYE detector was used for data collection. Diffraction data was collected from 10° to 70° 2θ with a 0.015° step size. Figure 4 shows powder XRD patterns for samples obtained after different times of heating at 170 °C. ZnO microstructures obtained after 2 and 10 min of reaction time can be indexed to wurzite ZnO (JCPDS # 01-079-2205) (Figure 4A,B, respectively). However, microstructures obtained after 20 min of heating have peaks corresponding to hydrated zinc silicate hydroxide (Zn_2_Si_2_O_7_·H_2_O) (JCPDS# 01-075-1320) in addition to wurzite ZnO (Figure 4C).

### 3.2. Mechanism of ZnO Microstructure Generation

In convectional-heating methods, the growth mechanism for ZnO microstructures is determined by the initial precipitation (nucleation) phase. It is known that Zn^2+^ ions react with the OH− ions by two reversible competing mechanisms:

(1) $${\text{Zn}}^{2+}+{\text{OH}}^{-}\;⇋\text{ ZnO}↓\;+{\text{ H}}^{+}$$

(2) $${\text{Zn}}^{2+}+2{\text{OH}}^{-}\;⇋\text{ Zn}{\left(\text{OH}\right)}_{2}↓$$

In case of direct ZnO precipitation, which is to be expected in a system consisting of an equimolar mixture of HMT and Zn(NO_3_)_2_ heated to a temperature of 170 °C [19], crystal growth is thought to occur by nanoparticle aggregation: organized growth of the ZnO crystal by assembly of nanoparticles prominently along the *c*-axis of the wurzite crystal [19,21,22]. Consistent with this, on a HMT-Zn^2+^ system under neutral pH conditions and hydrothermal parameters, Vergѐs *et al.* have reported evidence showing that ZnO nanoparticles attach amongst themselves to form large microstructures over time [23]. The tripod and tetrapod microstructures observed in various samples at all time points of the reaction in this work are of typical occurrence for ZnO microstructures generated by direct ZnO precipitation. Direct ZnO precipitation from an aqueous solution promotes twinning, growth of multiple lattices from a common junction with individual crystals growing along their *c*-axes tetrahedral to each other, [24] and leads to generation of tripod and tetrapod microstructures repeatedly observed during the various stages of the reaction.

For convectional-heated chemical baths, Govender *et al.* have reported the presence of a kinetically-controlled dissolution-recrystallization ripening process which follows the nucleation and growth during later stages of a solution-phase reaction [6]. Such a phase, considered to be as a part of a ripening mechanism occurring due to consumption of initial reagents, occurs via dissolution of ZnO crystal back in the solution as Zn(OH)_2_ which subsequently recrystallizes to form new 1D ZnO microrods. The morphological evolution of ZnO microstructures observed in Figure 1, leading to generation of a high proportion of ZnO microrods, could be attributed to an accelerated cyclic dissolution-recrystallization process made possible by localized molecular heating of the reaction mixture in the microwave cavity.

Further evidence for a dissolution-recrystallization mechanism comes from the presence of Zn silicates in the caps of ZnO microstructures, seen in samples after 20 min of microwave heating. Zinc silicate hydroxide (a.k.a. hemimorphite) can form by the action of Si(OH)_4_ on Zn(OH)_2_ as following [25,26]:

(3) $$\text{Zn}{\left(\text{OH}\right)}_{2}+\text{Si}{\left(\text{OH}\right)}_{4}\to {\text{Zn}}_{2}{\text{Si}}_{2}{\text{O}}_{7}\cdot {\text{H}}_{2}\text{O}↓$$

**Figure 5:**
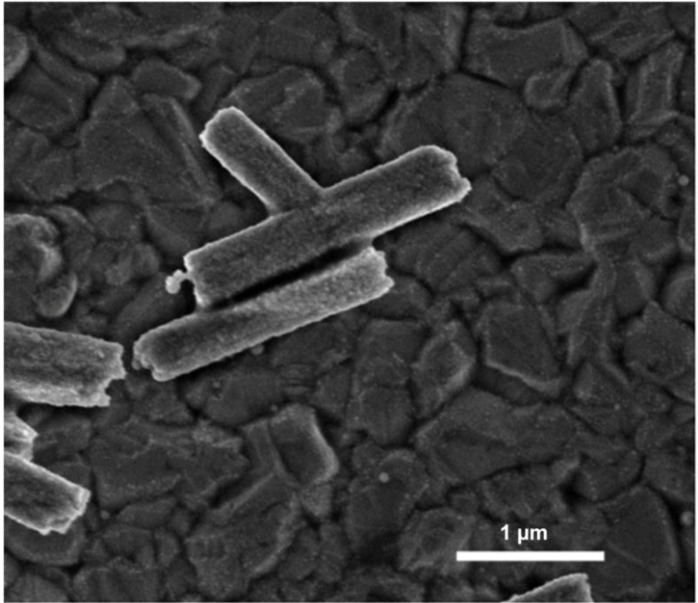
SEM image of ZnO microstructures synthesized by 25 mM equimolar mixture of Zn(NO_3_)_2_ and HMT at 100 °C for a period of 20 min.

The glass test tubes used for heating Zn^2+^ and HMT precursors in the single-mode microwave cavity in the CEM Discover^®^ system used here can serve as a source of Si(OH)_4_ up to 900 ppm (8.3 mM) at 200 °C [27,28]. Thus, a gradual formation of caps seen on rods and tripods could be due to a reaction between the Si(OH)_4_ impurities and Zn(OH)_2_ which is formed due to the dissolution-recrystallization process. Ideally, the formation of such zinc silicates is undesirable and can be avoided by using lower reaction temperatures. Figure 5 shows ZnO microrods and tripods generated by a 25 mM equimolar mixture of Zn(NO_3_)_2_ and HMT at 100 °C. At this temperature, Si(OH)_4_ concentration should be less than 500 ppm (4.6 mM) which should reduce or eliminate cap formation, as observed in Figure 5. The formation of zinc silicate hydroxide during the later stages of the reaction at higher temperatures of 170 °C serves as a marker to indicate the presence of Zn(OH)_2_ and provides evidence for the dissolution-recrystallization mechanism.

**Figure 6:**
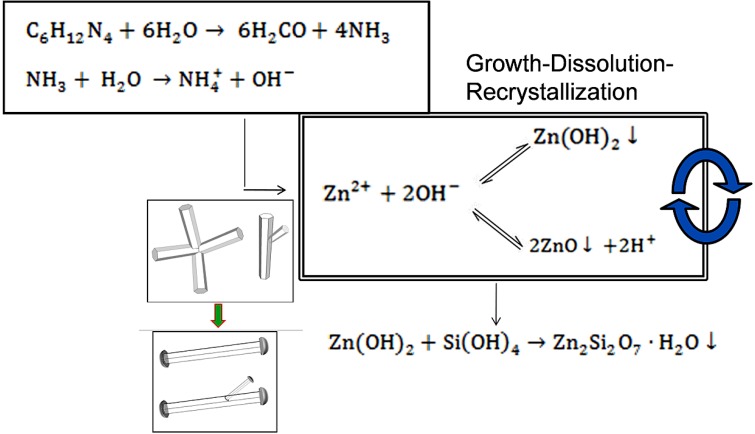
Schematic diagram showing the reaction mechanism of generation of ZnO microstructures generated by microwave-assisted heating.

Figure 6 shows a schematic representing the growth mechanism that occurs in the microwave-assisted hydrothermal process for synthesis of ZnO microstructures. Twinned ZnO microstructures, in the form of tetrapods and tripods, are initially formed by direct ZnO precipitation. Subsequently, ZnO microstructures undergo a reversible dissolution and recrystallization process via formation of Zn(OH)_2,_ leading to formation of one dimensional (1D) ZnO microrods.

**Figure 7:**
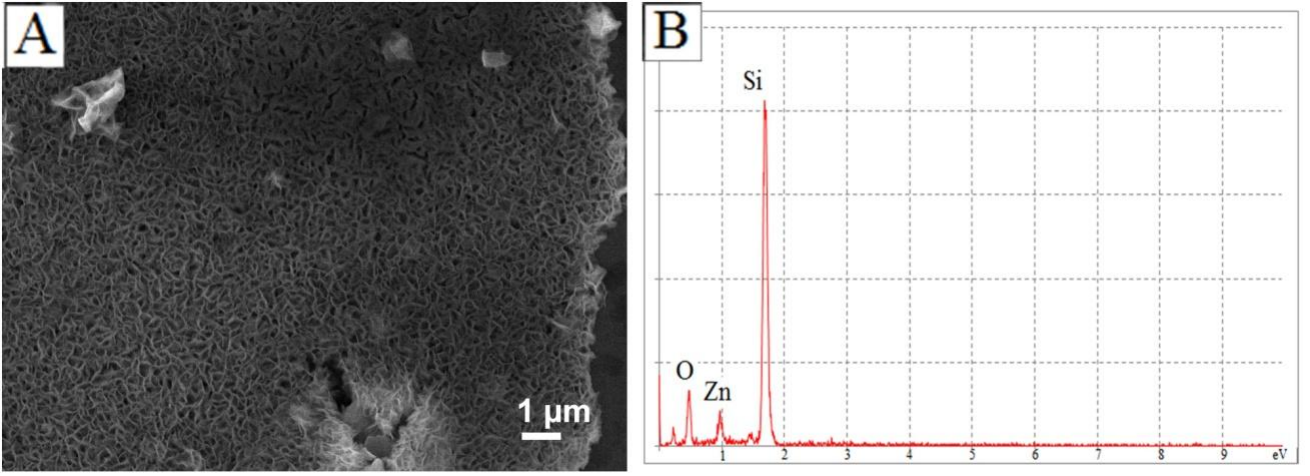
(**A**) SEM image of zinc silicate films generated after a period of three hours with microwave-assisted heating; (**B**) EDS confirms the chemical composition of the films.

In the system discussed here, irreversible formation of zinc silicate hydroxide effectively competes with and consequently disallows the recrystallization process from Zn(OH)_2_ to ZnO. Therefore, if the reaction were to proceed for a long duration of time, all ZnO microstructures should convert to zinc silicates via Zn(OH)_2_ formation. This indeed happens when the reaction is allowed to continue for a period of three hours. At three hours all ZnO microstructures are converted into films, as observed in Figure 7A, which consist of zinc silicates, as confirmed by EDS shown in Figure 7B.

## 4. Conclusions

In this study, synthesis of high quality ZnO microstructures by means of a microwave-assisted hydrothermal process is demonstrated, and the reaction mechanism for the growth of ZnO microstructures is analyzed. An accelerated rate of reaction obtained using microwaves lends to a morphological evolution of ZnO microstructures in a very short reaction time span. A dissolution-recrystallization mechanism dictates the generation of 1D ZnO microrods (and tripods) via formation of Zn(OH)_2_. Results presented in this work also show presence of zinc silicate caps on ZnO microstructures. In the current system, longer reaction times could be used for the synthesis of nanofilms of hemimorphite (zinc silicate hydroxide), a material with interesting optoelectronic properties. The generation of zinc silicates can be avoided at lower temperatures (~100 °C). However, high-quality faceted ZnO microstructures were generated only at higher temperatures of 170 °C.

## Supplementary Files

Supplementary File 1
