# Supplementary material for: Mechanism of Generation of ZnO Microstructures by Microwave-Assisted Hydrothermal Approach
Source: Materials (Basel). 2013 Jun 18;6(6):2497–507. doi: 10.3390/ma6062497 (PMC5458951; doi:10.3390/ma6062497)
Supplement: Supplementary File 1 [file materials-06-02497-s001.pdf]

Materials **2013**, *6*, 2497-2507; doi:10.3390/ma6062497

OPEN ACCESS

materials

ISSN 1996-1944

www.mdpi.com/journal/materials

Article

Figure S1. Temperature profile of the reaction system during synthesis at of ZnO microrods (Hold temp: 170 ℃, Hold time 20 min).

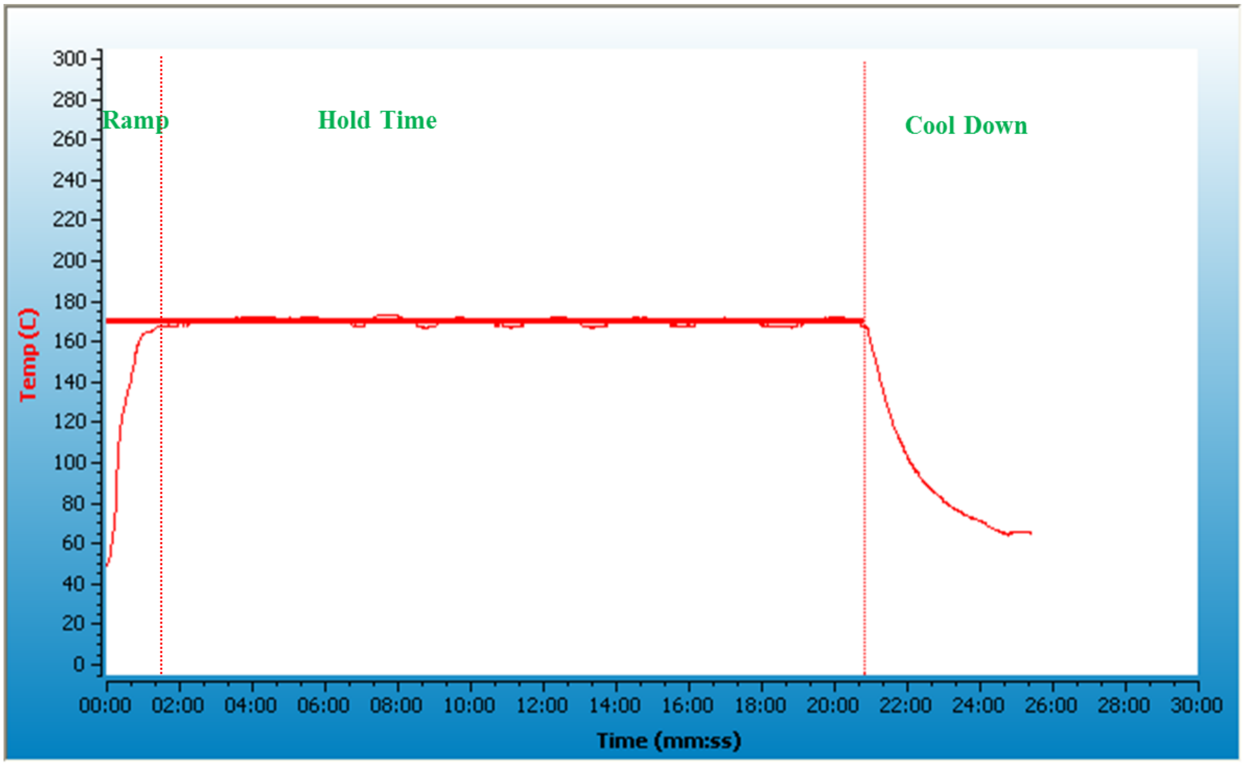

© 2013 by the authors; licensee MDPI, Basel, Switzerland. This article is an open access article distributed under the terms and conditions of the Creative Commons Attribution license (http://creativecommons.org/licenses/by/3.0/)
